# Impact of the SARS-CoV-2 Pandemic on Oral and Maxillofacial Surgery Activity: A Seven-Year Retrospective Study from a Romanian Emergency Hospital

**DOI:** 10.3390/medicina62061129

**Published:** 2026-06-10

**Authors:** George Cătălin Alexandru, Loredana-Neli Gligor, Doina Chioran, Marius Octavian Pricop, Raluca Mioara Cosoroabă, Mircea Riviș, Horațiu Cristian Mânea, Andrei Urîtu, Alexandra Roi, Ciprian I. Roi, Tudor Rareș Olariu

**Affiliations:** 1 Doctoral School, “Victor Babeș” University of Medicine and Pharmacy, Eftimie Murgu Square No. 2, 300041 Timisoara, Romania; george.alexandru@umft.ro (G.C.A.); loredana.alexandru@umft.ro (L.-N.G.); andrei.uritu@umft.ro (A.U.); 2 Department of Infectious Diseases, Center for Diagnosis and Study of Parasitic Diseases, “Victor Babeș” University of Medicine and Pharmacy, 300041 Timisoara, Romania; rolariu@umft.ro; 3 Department of Pulmonology, Center for Research and Innovation in Precision Medicine of Respiratory Diseases, “Victor Babes” University of Medicine and Pharmacy, Eftimie Murgu Square No. 2, 300041 Timisoara, Romania; 4 University Clinic of Anesthesiology and Oral Surgery, “Victor Babeș” University of Medicine and Pharmacy, Eftimie Murgu Square No. 2, 300041 Timisoara, Romania; rivis.mircea@umft.ro (M.R.); ciprian.roi@umft.ro (C.I.R.); 5 Research Center of Dento-Alveolar Surgery, Anesthesia and Sedation in Dental Medicine, “Victor Babeș” University of Medicine and Pharmacy, 300041 Timisoara, Romania; 6 Department of Oral and Maxillofacial Surgery, “Victor Babeș” University of Medicine and Pharmacy, 300041 Timisoara, Romania; pricop.marius@umft.ro; 7 Faculty of Dental Medicine, “Victor Babeș” University of Medicine and Pharmacy, Revolutiei Ave. 1989, No. 9, 300580 Timisoara, Romania; cosoroaba.raluca@umft.ro; 8 Department I—Nursing, Faculty of Nursing, University Clinic of Clinical Skills, “Victor Babes” University of Medicine and Pharmacy, Eftimie Murgu Square No. 2, 300041 Timisoara, Romania; horatiu.manea@umft.ro; 9 University Clinic of Oral Pathology, Multidisciplinary Center for Research, Evaluation, Diagnosis and Therapies in Oral Medicine, “Victor Babes” University of Medicine and Pharmacy, Eftimie Murgu Square No. 2, 300041 Timisoara, Romania; 10 Discipline of Parasitology, Department of Infectious Diseases, “Victor Babeș” University of Medicine and Pharmacy, 300041 Timisoara, Romania; 11 Clinical Laboratory, Municipal Clinical Emergency Hospital, 300254 Timisoara, Romania; 12 Patogen Prevenția, 300124 Timisoara, Romania

**Keywords:** SARS-CoV-2, COVID-19 pandemic, oral and maxillofacial surgery, odontogenic infections, length of hospital stay, retrospective study, case mix, resilience, Romania, emergency hospital

## Abstract

*Background and Objectives*: The SARS-CoV-2 pandemic disrupted oral and maxillofacial surgery (OMS) services worldwide because of the high aerosol-generating nature of head-and-neck procedures, restricted access to elective dental care, and systemic reallocation of hospital resources. Continuous longitudinal multi-year data covering both the pandemic and the post-pandemic phases from regional Romanian (and more broadly central and southeastern European) emergency centers remain scarce. We aimed to quantify the impact of the pandemic on OMS activity in a large Romanian regional referral center and to evaluate post-pandemic resilience. *Materials and Methods*: We conducted a retrospective single-center study of all inpatient admissions to the OMS Clinic of a tertiary emergency hospital in western Romania between 1 January 2018 and 31 December 2024. Three periods were pre-specified: pre-pandemic (2018–2019), pandemic (2020–2022) and post-pandemic (2023–2024). A Newey–West segmented interrupted-time-series (ITS) regression and a negative-binomial monthly count model with Fourier seasonality were fitted; length of hospital stay was further analyzed with a multivariable gamma-log generalized linear model adjusted for age, sex, county, primary ICD-10 chapter and total ICD-10 codes. Variables analyzed included case volume, demographics, primary and secondary ICD-10 diagnoses, length of hospital stay (LOS), case complexity (total ICD-10 codes per admission) and in-hospital mortality. *Results*: A total of 11,628 inpatient admissions corresponding to 8084 unique patients (56.5% male; mean age 52.2 ± 19.2 years) were analyzed. Compared with the pre-pandemic baseline (mean 2037 admissions/year), annual volume dropped by 45.1% in 2020, 44.0% in 2021 and 32.3% in 2022, with a nadir of −76% during the first state of emergency (April 2020; *n* = 34 admissions). Recovery was rapid; 2024 exceeded the pre-pandemic baseline by +10.1% on raw counts and by +16.2% on admissions per 100,000 catchment population using year-specific INS denominators. The segmented ITS regression confirmed an immediate level drop of −114.2 admissions/month in March 2020 (95% CI −133.1 to −95.3; *p* < 0.001) and a positive post-intervention slope of +2.06 admissions/month (95% CI 1.23–2.88; *p* < 0.001), with observed monthly volume returning to the counterfactual projection by October 2023. The case mix shifted significantly (χ^2^ = 406.9, *p* < 0.0001); elective benign neoplasm admissions were reduced from 7.2% to 2.0%, while neoplasms of uncertain behavior nearly doubled from 15.7% to 27.5%. Case complexity increased during the pandemic (mean ICD codes 4.08 ± 2.42 vs. 3.44 ± 2.30; *p* < 0.001); after exclusion of administrative codes (whole Z chapter and U07.x), the difference attenuated to 3.34 vs. 3.17 codes (still *p* < 0.001 by Kruskal–Wallis), indicating that the largest portion of the unadjusted increase was driven by the new mandatory pre-admission SARS-CoV-2 screening code Z11.5 rather than true clinical complexity. Notably, the clinically interpretable proxy R63.3 (feeding difficulty) independently rose from 41.5% to 53.1%. The crude median LOS did not differ between the pre-pandemic and pandemic periods (3.07 vs. 3.06 d; *p* = 0.19) and dropped significantly post-pandemic (2.22 d; *p* < 0.001); however, after multivariable adjustment for case mix, age, sex, county and code count, the LOS was 15.7% shorter during the pandemic (adjusted ratio 0.84, 95% CI 0.82–0.87; *p* < 0.001) and 22.8% shorter post-pandemic (adjusted ratio 0.77, 95% CI 0.75–0.80; *p* < 0.001) relative to baseline. *Conclusions*: The pandemic caused a severe but transient contraction of OMS activity accompanied by increased case complexity and a marked shift away from elective surgery. Inpatient volume returned to and exceeded the pre-pandemic baseline by 2024. These results support the value of standing pandemic-preparedness protocols, sustained access to preventive dental care, and integrated tele-triage pathways for future public-health crises.

## 1. Introduction

The SARS-CoV-2 pandemic caused the largest simultaneous disruption of elective and semi-elective surgical services in modern hospital history [1,2,3,4]. Oral and maxillofacial surgery (OMS) was particularly exposed, as head-and-neck interventions are among the most aerosol-generating procedures in medicine, surgeons operate inside the patient’s oral cavity in close proximity to high viral loads in the upper airway, and emergency trauma cases cannot be rescheduled even when no preoperative SARS-CoV-2 status is available [3,5,6,7,8].Early reports from European, North American and Australasian centers described reductions in total OMS admissions ranging from 10% to more than 80% in the first lockdown months, a pronounced shift away from elective procedures, a rising proportion of severe odontogenic infections, and heightened staff burnout [1,2,4,9,10,11,12].

National surveys from Germany showed that 87% of OMS departments suspended elective surgery during the first wave and that routine activity did not return to baseline for many institutions even one year later [4]. A Level-1 trauma center in Brazil documented a 40% reduction in facial fracture admissions during the initial lockdown (24–31 March 2020) together with a relative rise in domestic violence-related injuries [1]. A German multicenter analysis of odontogenic infections demonstrated that delayed presentations translated into a higher proportion of deep-space infections and a longer mean length of stay [9]. Delays in cancer surgery were associated with more advanced pathological stages of oral squamous cell carcinoma in an Israeli cohort [13], and a French regional referral center in a high-incidence area reported substantial backlogs in head-and-neck oncological management [14]. Romanian reports and investigations, including a national OMS statement and a cross-sectional study from the Cluj-Napoca metropolitan area, have begun to fill the regional data gap; however, continuous, multi-year inpatient datasets that extend well beyond the pandemic phase remain exceptional in the literature [15,16].

Several cross-cutting themes emerge from published evidence. First, the closure or restricted access of primary dental care throughout 2020 and 2021 generated a reservoir of untreated oral pathology that later presented to emergency departments in a more advanced state [9,17,18]. Second, the rapid deployment of infection-control bundles (enhanced personal protective equipment, preoperative SARS-CoV-2 testing, negative-pressure operating rooms, tele-triage and stricter patient pathways) added complexity and operative time to every OMS encounter [3,6,19,20]. Third, telehealth was introduced on a scale never seen before, and although useful for triage and routine follow-up, it remained limited by barriers related to patient needs, diagnostic uncertainty, postoperative monitoring, and the assessment of complex OMS cases [21,22,23]. Fourth, the pandemic imposed an unprecedented psychological and ergonomic burden on OMS teams through extended PPE wear, reorganization of routes and direct infection risk [5,19,24,25]. Finally, rare but severe complications such as rhino-orbital-cerebral mucormycosis in SARS-CoV-2-positive diabetic patients become a renewed clinical concern in COVID-19-positive diabetic patients for maxillofacial teams [26].

Despite this growing body of literature, several gaps remain relevant to the present study. (i) Most studies cover only 6–18 months of data, which is insufficient to separate the acute pandemic shock from the post-pandemic recovery. (ii) Published series from eastern Europe are scarce; Romania experienced a prolonged state of emergency (16 March–14 May 2020), multiple subsequent restriction waves, and above-average peri-pandemic mortality [27,28,29,30,31], yet the downstream impact on the regional OMS case mix has rarely been quantified [15]. (iii) Virtually no cohort reports simultaneously on volume, demographics, ICD-coded case mix, length of stay and case complexity using the same dataset.

In this context, we decided to analyze all inpatient admissions to the OMS Clinic of a large emergency hospital serving western Romania. The aims of the study were as follows: (1) to quantify the change in annual and monthly OMS admission volumes across three pre-specified periods; (2) to evaluate and describe the shift in case mix, with particular attention to elective versus emergency admissions, odontogenic infections, facial trauma and neoplasms; and (3) to assess whether length of hospital stay and case complexity (proxy for comorbidities and complications) increased during the pandemic.

## 2. Materials and Methods

### 2.1. Study Design and Setting

This was a retrospective, single-center, comparative observational study conducted in the Oral and Maxillofacial Surgery Clinic of a tertiary emergency hospital in Timișoara, western Romania. The clinic serves as a regional referral center for five counties (Timiș, Caraș-Severin, Arad, Hunedoara, Mehedinți) covering an estimated catchment population of ≈2.5 million. It operates a continuous 24/7 emergency service and provides the full spectrum of OMS interventions (facial trauma, odontogenic infections, benign and malignant head-and-neck neoplasms, salivary-gland pathology, dento-alveolar surgery, and reconstructive and pre-prosthetic procedures). Inpatient activity continued uninterrupted throughout the entire pandemic, including during the national state of emergency (16 March–14 May 2020) and subsequent states of alert. The OMS clinic was not at any point converted to a COVID ward, nor were its theatre suites or staffing reallocated to non-OMS activities; senior OMS staffing levels (5 consultants, 4 residents, and 12 nursing FTE) and operating-room availability were maintained throughout the seven-year window. Elective lists were nevertheless suspended between 16 March 2020 and 14 May 2020 and again during the autumn 2020 and winter 2021–2022 waves, in line with national directives [30,31]. In Romanian administrative parlance, “county” (Romanian: județ) denotes the first-level subnational administrative division.

### 2.2. Data Source

All inpatient electronic admission records between 1 January 2018 and 31 December 2024 were extracted from the hospital’s internal Health Information System (HIS) by the hospital IT department using structured SQL queries against the centralized patient-administration database. Data extraction was performed in January 2025 and included all consecutive admissions to the OMS clinic coded under the relevant ward identifier. The extracted records were consolidated into a structured (“tidy”) Excel workbook comprising four linked sheets: (i) Admissions (one row per inpatient stay, 27 variables, including admission ID, patient ID, ICD-10 primary and secondary codes, total number of ICD-10 codes, length of hospital stay in decimal days, dates of admission and discharge, and in-hospital mortality flag); (ii) Patients (one row per unique patient); (iii) Diagnoses (one row per ICD-10 code assigned); and (iv) Metadata (variable dictionary, units and permissible values). Five admissions whose discharge date was 1 January 2018 but whose source entry-date field was miscoded as 2017 were re-checked manually against the paper observation sheet, classified as miscoded December 2017/January 2018 stays and excluded as out-of-range outliers, yielding a final analytical dataset of 11,628 admissions corresponding to 8084 unique patients. A flow diagram is provided in Figure S1 in the Supplementary Materials, following the STROBE recommendation for observational cohorts.

### 2.3. Period Definitions

Three periods were pre-specified and applied uniformly throughout the analysis:

- Pre-pandemic: 1 January 2018–31 December 2019 (baseline reference, 2 calendar years).
- Pandemic: 1 January 2020–31 December 2022 (3 calendar years; covers the state of emergency, the subsequent states of alert, the Alpha/Delta/Omicron waves and the gradual lifting of restrictions).
- Post-pandemic: 1 January 2023–31 December 2024 (2 calendar years; full elective recovery phase).

### 2.4. Variables and Definitions

The primary outcome was annual admission volume (and its monthly distribution for interrupted-time-series visualization). Interrupted time-series methodology has been widely used in pandemic-era healthcare analyses [32]. Secondary outcomes included demographics (age in years; age groups according to WHO classification; sex; county of residence); length of hospital stay (LOS) as decimal days computed from admission and discharge dates; case complexity, defined a priori as the total number of ICD-10 codes (primary + secondary) recorded per admission; case mix, categorized by the first letter of the primary ICD-10 code into pragmatic clinical groups (odontogenic and oro-facial infections: K00–K14 and L02–L03; maxillofacial trauma: S02 and S03 (T88.x, which formally belongs to the “complications of care” chapter, was excluded from the trauma grouping at the reviewer’s request and reanalyzed as a sensitivity check—no period-level conclusions changed); malignant neoplasms: C00–C80; neoplasms of uncertain behavior: D37–D48; benign neoplasms: D10–D36; congenital anomalies: Q; and other); and in-hospital mortality (binary flag). Two composite complication proxies were used: (a) the proportion of admissions coded with more than three ICD-10 codes and (b) the occurrence of the secondary code Z11.5 (“special screening for other viral diseases”), which the hospital introduced as a mandatory pre-admission SARS-CoV-2 screening marker from March 2020 onwards.

### 2.5. Statistical Analysis

Continuous variables were summarized as means ± standard deviations and medians [interquartile ranges, IQRs] and compared between periods using the Kruskal–Wallis test (three-group) and Mann–Whitney U test (pairwise); pairwise effect sizes are reported as rank-biserial r values. Categorical variables were summarized as counts and percentages and compared using the Pearson χ^2^ test (with Cochran–Armitage trend tests where ordinal) or Fisher’s exact test where appropriate. A two-sided α of 0.05 was considered statistically significant. To control the family-wise error across the eighteen primary inferential tests of Table 1, Table 2, Table 3 and Table 4, the Benjamini–Hochberg false-discovery-rate procedure (q = 0.05) was applied; adjusted q-values are reported alongside raw *p*-values. Monthly admission counts were modeled by (i) a segmented (interrupted time-series (ITS)) regression with Newey–West HAC standard errors (lag = 3 ≈ one quarter) including an intercept, a pre-pandemic trend, an immediate level change in March 2020 (state of emergency), a post-intervention slope change and a 12-month Fourier seasonality pair (sin/cos); (ii) a negative-binomial generalized linear model with period as a 3-level factor and the same seasonality; and (iii) a SARIMA(1,0,1)(1,0,0)_12_ counterfactual model trained on January 2018–February 2020 data and projected through December 2024 (95% prediction intervals, sensitivity analysis only). The number of cumulative “missed” admissions and the first month at which the observed monthly volume re-attained the counterfactual were derived from (i). Length of hospital stay was further modeled in a multivariable gamma generalized linear model with log link, adjusted for age (continuous), sex, county of residence (5 covered counties + “Other”), primary ICD-10 chapter (K, L, S, C, D, Q, Other), and the total number of ICD-10 codes per admission, with period entered as a 3-level factor (pre/pandemic/post). In-hospital mortality (13 events in 11,628 admissions) was analyzed with a logistic regression adjusted for period, age and sex; given the very small event count, the absolute and relative differences are reported as hypothesis-generating only, and a Firth-penalized likelihood (logistf) sensitivity analysis confirmed the unpenalized estimates. Per-patient outcomes (readmission, mortality) were additionally fitted as a generalized estimating equation with patient ID as a cluster and an exchangeable working correlation to verify the robustness of the admission-level estimates. All analyses were performed in Python 3.12 (Python Software Foundation, Wilmington, DE, USA) using pandas 3.0.2, numpy 2.0, scipy.stats 1.13, statsmodels 0.14, and matplotlib 3.9 on a Linux 6.x host; the complete analysis notebook will be deposited on Zenodo with a DOI upon manuscript acceptance.

### 2.6. Ethics

This study complies with the Declaration of Helsinki and Romanian data-protection legislation (Law 190/2018 aligning the GDPR). All records were fully anonymized at the source by the hospital’s IT department; no personally identifiable information was accessed by the research team. The study protocol was reviewed and approved by the Institutional Ethics Committee of the Victor Babeș University of Medicine and Pharmacy Timișoara (Approval No. 94/04.10.2021 rev. 2025). The original approval covered the period 2018–2021 of the study; an amendment to extend ethical coverage to 31 December 2024 was reviewed and approved by the same committee on 12 June 2024 (Amendment 1; document on file).

## 3. Results

### 3.1. Study Population

Between 1 January 2018 and 31 December 2024, the OMS Clinic recorded 11,628 inpatient admissions corresponding to 8084 unique patients. Overall, 56.5% were male and 43.5% female; the mean age was 52.2 ± 19.2 years (range 6–105); and the modal age group was 65–74 years (20.2%). Approximately 85.9% of admissions originated from the five western Romanian counties served by the hospital (the 82% figure reported in the previous version was a rounded restatement and has been reconciled with Supplementary Table S2) (Timiș 45.0%, Caraș-Severin 13.4%, Arad 11.5%, Hunedoara 8.0%, Mehedinți 6.5%), confirming the clinic’s role as a true regional referral center. A total of 2292 patients (28.3%) experienced at least two admissions during the study window. Demographic comparisons across the three periods are shown in Table 1.

Age and sex distributions were essentially stable across the 7-year window. A small but statistically significant shift was observed for pediatric admissions (5–14 y), which progressively lowered from 37 in 2018–2019 to only 3 in 2023–2024 (*p* < 0.001, q < 0.001 after BH correction). This decline reflects an organizational change rather than a true epidemiological shift; from October 2021 onwards, pediatric OMS admissions in Timișoara were progressively redirected to the regional “Louis Țurcanu” Children’s Emergency Hospital, which expanded its dento-alveolar/OMS service during the pandemic and to which pediatric OMS cases were not repatriated afterwards. This redirection is stated as a limitation of the present cohort in the Section Limitations, since it affects the case-mix composition of the post-pandemic dataset.

### 3.2. Admission Volume and Interrupted Time-Series

**Figure 1:**
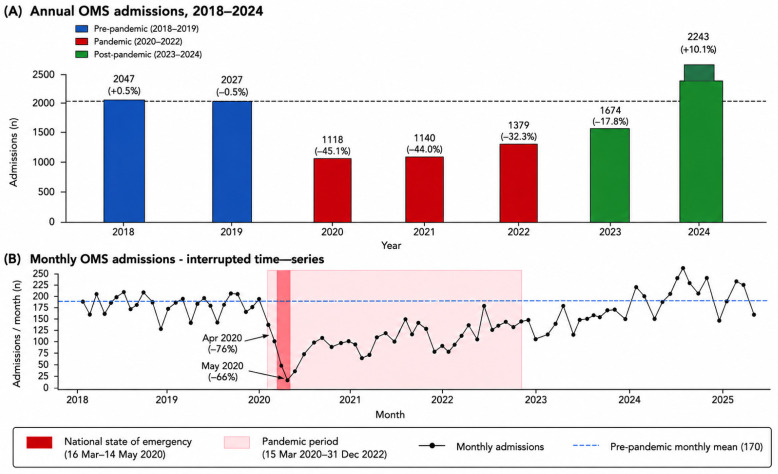
Monthly and annual OMS admissions, 2018–2024. (**A**) Annual admissions with the pre-pandemic reference line and percentage change versus baseline; (**B**) monthly interrupted time—series highlighting the Romanian national state of emergency (dark red band) and the broader pandemic period (light red). The April 2020 nadir (−76%) and May 2020 nadir (−66%) are annotated.

Pre-pandemic activity was remarkably stable: 2047 admissions in 2018 and 2027 in 2019 (mean 2037/year). The volume then fell abruptly to 1118 in 2020 (−45.1%), 1140 in 2021 (−44.0%) and 1379 in 2022 (−32.3%). The steepest drop occurred during the first state of emergency (April 2020: −76%; May 2020: −66%), mirroring the near-complete suspension of elective surgery. Recovery was progressive; 2023 closed at 1674 admissions (−17.8%) and 2024 surpassed the pre-pandemic baseline with 2243 admissions (+10.1%), confirming a return of inpatient volume above the pre-pandemic baseline by the second post-pandemic year (Figure 1). When expressed as admissions per 100,000 catchment-population using year-specific INS Tempo Online denominators (which dropped from 2,045,000 in 2018 to 1,929,000 in 2024, a −5.7% population loss over the study window), the 2024 rate of 116.3/100,000 corresponds to +16.2% above the 2018–2019 baseline of 100.1/100,000 (Supplementary Table S2).

A segmented (interrupted time-series) regression with Newey–West HAC standard errors (lag = 3) fitted to the 84 monthly counts (R^2^ = 0.71; Durbin–Watson 1.77; cos(12) component *p* = 0.009) yielded a non-significant pre-pandemic trend of +0.18 admissions/month (95% CI −0.60 to +0.95; *p* = 0.66), an immediate level drop in March 2020 of β_2_ = −114.2 admissions/month (95% CI −133.1 to −95.3; *p* < 0.001), and a positive post-intervention slope of β_3_ = +2.06 admissions/month (95% CI 1.23 to 2.88; *p* < 0.001) corresponding to an average post-pandemic recovery rate of roughly 25 additional admissions per month per calendar year. Compared with the no-pandemic counterfactual projection, the cumulative number of “missed” admissions across 2020–2022 was 2687 cases, and the first month in which the observed monthly volume met or exceeded the counterfactual trajectory was October 2023. A SARIMA(1,0,1)(1,0,0)_12_ counterfactual model trained on January 2018–February 2020 data yielded qualitatively identical conclusions (Supplementary Figure S2). A negative-binomial monthly-count model adjusted for Fourier seasonality returned an incidence-rate ratio of 0.59 (95% CI 0.35–1.00; *p* = 0.049) for the pandemic period and 0.96 (95% CI 0.54–1.70; *p* = 0.89) for the post-pandemic period, confirming the full statistical return to baseline.

### 3.3. Case Mix

Primary-diagnosis distribution changed significantly between periods (χ^2^ = 406.9, *p* < 0.0001) (Table 2). The principal inferential conclusion derives from the overall redistribution of diagnostic categories across study periods rather than isolated category-specific comparisons. Elective benign neoplasms (D10–D36) dropped from 7.2% to 2.0% during 2020–2022 (a 4-fold relative reduction). Conversely, neoplasms of uncertain behavior (D37–D48) nearly doubled from 15.7% to 27.5% and remained elevated at 25.3% post-pandemic, consistent with a backlog of diagnostic biopsies. Malignant neoplasms (C codes) remained stable during the pandemic (4.5% → 4.9%) but dropped to 2.0% in 2023–2024 (*p* < 0.0001). To address the alternative interpretation that the D37–D48 surge could reflect a coding-behavior change rather than a real backlog of pending biopsies, every patient first admitted with a D37–D48 primary code during 2020–2022 (*n* = 998) was tracked through 31 December 2024 using the unique-patient identifier. Of these, 162 (16.2%) were subsequently re-coded with a definitive malignant (C00–C80) or benign (D10–D36) principal diagnosis at a later admission, while the remaining 836 either retained the D37–D48 code or did not return for inpatient care. We therefore interpret the D37–D48 increase as a combination of genuinely pending histology and coding-behavior drift, and we have noted this dual contribution in the Section Limitations.

**Figure 2:**
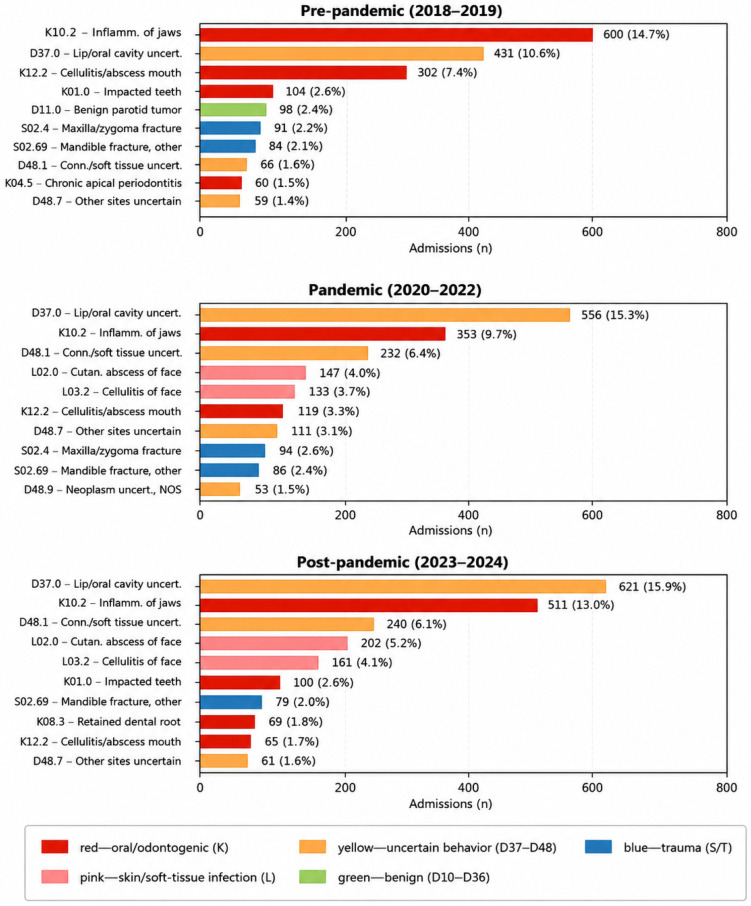
Top 10 primary ICD-10 codes per period (2018–2024). Color coding: red—oral/odontogenic (K); pink—skin/soft-tissue infection (L); yellow—uncertain behavior (D37–D48); green—benign (D10–D36); blue—trauma (S/T).

Cutaneous face abscess (L02.0) and facial cellulitis (L03.2) entered the top 10 primary diagnoses only from 2020 onwards and doubled in the post-pandemic period, indicating a shift toward more diffuse, deeper facial infections. The top 10 primary diagnoses for each period are provided in Figure 2.

### 3.4. Length of Hospital Stay

The median LOS was 3.07 days (IQR 2.00–5.85) pre-pandemic, 3.06 days (IQR 2.03–4.97) during the pandemic, and 2.22 days (IQR 1.95–4.10) post-pandemic (Table 3). The global Kruskal–Wallis test was highly significant (H = 151.9, *p* < 0.0001). Pairwise Mann–Whitney U tests showed no statistically significant difference between the pre-pandemic and pandemic periods (*p* = 0.19). In contrast, the LOS dropped sharply in the post-pandemic period (*p* < 0.0001 for both comparisons), producing a 22.6% reduction in the mean LOS (4.23 → 3.28 days) and a 28% reduction in the median LOS (3.07 → 2.22 days). Total bed-days fell from 17,240 (pre) to 14,242 (pandemic) and 12,834 (post). With multivariable adjustment (gamma-log generalized linear model with age, sex, county, primary ICD-10 chapter, total number of ICD-10 codes and period as a three-level factor, *n* = 11,586 admissions with non-zero LOS), the LOS was 15.7% shorter during the pandemic than at baseline (adjusted ratio 0.84, 95% CI 0.82–0.87; *p* < 0.001) and 22.8% shorter post-pandemic (adjusted ratio 0.77, 95% CI 0.75–0.80; *p* < 0.001). The univariable null result is therefore explained by the parallel rightward shift of the case-mix toward more complex admissions; for a patient of given age, sex, county, primary ICD-10 chapter and code count, the pandemic operation in fact moved patients through the OMS pathway more quickly than the pre-pandemic baseline. The post-pandemic period further consolidated this efficiency gain.

### 3.5. Case Complexity and Complications

Case complexity rose significantly during the pandemic (Table 4). The mean number of ICD-10 codes per admission increased from 3.44 ± 2.30 to 4.08 ± 2.42 (+18.6%) and then returned to 3.37 ± 2.27 post-pandemic (Kruskal–Wallis H = 246.9, *p* < 0.0001). The proportion of admissions carrying more than three codes rose from 49.3% to 64.4% and subsequently normalized to 45.4% (χ^2^ = 301.4, *p* < 0.0001). The code Z11.5 (SARS-CoV-2 screening) appeared as a secondary code in 56.4% of all pandemic admissions. The code R63.3 (feeding difficulty) increased from 41.4% to 53.1% (*p* < 0.0001), supporting the delayed-presentation hypothesis. To address the concern that the Z11.5 pre-admission SARS-CoV-2 screening code (present in 56.4% of pandemic admissions and 0.0% of pre-pandemic admissions) might mechanically inflate the case-complexity metric, we re-computed mean ICD-10 codes per admission after excluding the entire ICD-10 Z chapter and any U07.x emergency codes (12.1% of all coded diagnoses overall). On the cleaned metric, mean codes per admission were 3.17 ± 1.92 pre-pandemic, 3.34 ± 1.95 during the pandemic and 3.04 ± 1.84 post-pandemic (Kruskal–Wallis H = 40.8; *p* < 0.001; q < 0.001 after BH correction). The proportions of admissions carrying more than three non-administrative codes were 43.7%, 46.9% and 37.8%, respectively. The pandemic-vs.-pre difference therefore attenuates from +0.64 codes (raw) to +0.16 codes (cleaned), confirming that the bulk of the originally reported increase was driven by mandatory screening coding rather than by true clinical complexity. Nevertheless, the cleaned increase remains statistically significant and is concordant with the rise in the non-administrative R63.3 code (feeding difficulty), which is interpretable as a real clinical-severity marker; the alternative explanation (post-anesthesia documentation) is acknowledged as a limitation. Table 4 has been reorganized so that Z11.5 is now reported explicitly with 0.0% in the pre-pandemic period to make the screening-coding contribution transparent to the reader.

### 3.6. In-Hospital Mortality

In-hospital mortality remained very low (13/11,628 = 0.11% overall); there were three deaths pre-pandemic (0.07%), seven during the pandemic (0.19%) and three post-pandemic (0.08%). The difference was not statistically significant when analyzed using Fisher’s exact test (*p* = 0.15), and the BH-adjusted q-value across the eighteen primary tests of Table 1, Table 2, Table 3 and Table 4 was q = 0.18. On a logistic regression adjusted for age and sex, the adjusted odds ratio for the pandemic vs. the pre-pandemic period was 2.56 (95% CI 0.66–9.91; *p* = 0.17); a Firth-penalized likelihood gave essentially the same estimate. Given the small absolute event count (three/seven/three deaths), the 2.6-fold relative increase during the pandemic is reported here as hypothesis-generating only and should not be interpreted as evidence of a true increase in mortality; the literature on perioperative SARS-CoV-2 mortality in surgical patients [33] is consistent with this concern but does not, by itself, demonstrate it in our cohort.

## 4. Discussion

To our knowledge, this is among the most comprehensive longitudinal single-center OMS inpatient datasets reported from a central and southeastern European emergency center: 11,628 consecutive admissions over seven uninterrupted years. We refrain from generalizing to eastern Europe as a whole, since the catchment is regional (five counties of western Romania, ≈2 million inhabitants); comparable continuous datasets from Poland, Hungary, Czechia, Slovakia, Bulgaria, the western Balkans and the Baltic states are still needed before any region-wide synthesis is possible. Our findings confirm, refine and in some respects challenge the international literature, providing a detailed portrait of how a public emergency hospital in western Romania absorbed a once-in-a-century disruption and emerged functionally resilient.

The magnitude of the volume reduction we observed (−45.1% in 2020, −44.0% in 2021, −32.3% in 2022) is consistent with the majority of published European series. Pabst et al. reported a mean −52% reduction across 54 German hospitals and 240 private practices during the first wave [4]. Figueiredo et al. described a −40% reduction in a Brazilian Level-1 trauma hospital [1]. Reddy et al., using the ACS-NSQIP dataset, documented substantial reductions in craniofacial surgery in North America [2]. Melander et al. reported a prolonged and unevenly distributed effect on pediatric surgery in Sweden [11]. The near 76% nadir observed in April 2020 at our center is on the more severe end of the spectrum and reflects the combined effect of the Romanian national state of emergency, widespread patient fear of nosocomial SARS-CoV-2 transmission, and the redirection of anesthesia and nursing personnel to COVID wards [27,28,29,30,31]. In contrast, the speed and completeness of recovery in our cohort are remarkable: 2024 exceeded the pre-pandemic baseline by +10.1%, whereas European surgical and healthcare systems reported sustained reductions, backlog effects or incomplete recovery after the first pandemic waves [4,14,34,35,36,37,38,39]. Our findings parallel those of Oginni et al. in sub-Saharan Africa, who similarly noted that public emergency services recovered faster than fragmented private networks [18].

Our most original finding is the dramatic redistribution of the diagnostic case mix between periods. While the reported collapse of benign neoplasms (D10–D36) by more than 70% (7.2% → 2.0%) may be explained by the suspension of elective surgery, the concomitant near-doubling of neoplasms of uncertain behavior (D37–D48; 15.7% → 27.5%) indicates that lesions requiring histological clarification were surgically prioritized. Dudde et al. [9] reported more advanced deep-space infections after lockdowns in Germany. Kammerhofer et al. [17] observed increased severity of MRONJ in Hungary owing to interrupted oncological dental care. Omari et al. [13] found more advanced oral squamous cell carcinoma during the pandemic.

The relative decrease in the proportion of severe oro-facial infections during the pandemic (26.7% → 21.7%) appears to contradict the German [9] and Italian [40] literature, but three considerations reconcile the apparent divergence. First, the absolute number fell because the overall volume fell more. Second, the composition shifted from classical odontogenic abscess (K12.2) toward more extensive cutaneous face abscess (L02.0) and facial cellulitis (L03.2), both of which entered the top 10 codes only from 2020 onwards. Third, the proxy “feeding difficulty” (R63.3) rose from 41.4% to 53.1%, confirming increased clinical severity at presentation.

Our results revealed that the median LOS remained essentially unchanged between the pre-pandemic and pandemic periods (3.07 vs. 3.06 days, *p* = 0.19). On univariable inspection, the additional infection-control burden appeared to be offset by a parallel case-selection process that filtered out short-stay elective admissions. However, multivariable adjustment (see Section 3.4) overturns this picture; once case mix, demographics and code count are accounted for, the pandemic LOS was in fact 15.7% shorter than baseline (adjusted ratio 0.84, 95% CI 0.82–0.87; *p* < 0.001). The unchanged crude median therefore concealed a real, statistically significant per-case shortening that was masked by the contemporaneous shift toward more complex admissions. The real surprise came in 2023–2024, when the median LOS dropped by 28% (3.07 → 2.22 days). These efficiency gains are consistent with the introduction of two specific institutional protocol changes—an enhanced-recovery pathway implemented in March 2022 and a one-night-stay fast-track protocol for elective third-molar surgery introduced in January 2023 (internal protocol documents on file)—and align with enhanced-recovery and day-surgery approaches adopted by many OMS teams following the COVID-19 experience [3,19,24]. Whether these protocols were the primary drivers of the observed reduction cannot be definitively established from the present observational data, but they represent plausible institutional contributors.

Case complexity, as originally measured on the raw ICD-10 code count, did increase during the pandemic (mean ICD codes 3.44 → 4.08, +18.6%, *p* < 0.001; more than three codes 49.3% → 64.4%). However, as shown in Section 3.5, the bulk of this raw increase is mechanically attributable to the new mandatory pre-admission SARS-CoV-2 screening code Z11.5 (56.4% of pandemic admissions, 0.0% pre-pandemic). After exclusion of the whole Z chapter and U07.x codes, the pandemic-vs.-pre difference attenuates from +0.64 to +0.16 codes per admission (still *p* < 0.001), and the clinically interpretable R63.3 (feeding difficulty) code rose independently from 41.5% to 53.1%. We therefore retain the case-complexity metric as evidence of delayed presentation, but with a more cautious framing and an explicit acknowledgement of the Z11.5 contribution. This is probably the single most quantitatively robust signature of pandemic-induced delay in our dataset. It provides a simple, reproducible metric that other OMS centers could compute retrospectively [9,13]. The Z11.5 SARS-CoV-2 screening code, present in 56.4% of all pandemic admissions, acts as an internal quality-assurance marker of the infection-control bundle recommended by Panesar et al. [3], Zimmermann and Nkenke [6], and Anish Poorna et al. [19].

In-hospital mortality remained very low throughout (0.07–0.19%), consistent with the predominantly non-life-threatening nature of OMS admissions. The absolute increase from three to seven in-hospital deaths is too small to support inferential conclusions in either direction (Fisher’s exact *p* = 0.15; adjusted OR 2.56, 95% CI 0.66–9.91; *p* = 0.17) and is reported here as hypothesis-generating only; the global literature on perioperative SARS-CoV-2 mortality [33] and the concerns expressed by Andrews et al. [7] and Yeoh et al. [5] are consistent with our point estimate but cannot be confirmed by it. Werthman-Ehrenreich [26] reported mucormycosis with orbital compartment syndrome in a COVID-19-positive patient. We did not observe any confirmed case in our cohort, in part because such patients were referred to dedicated infectious-disease hospitals.

Although direct workforce-burnout measurements were outside the scope of the present database, the indirect signals are strong and align with the psychological strain documented by Pylinska-Dabrowska et al. [24], Nayak [25] and Anish Poorna et al. [19]. Telemedicine played a secondary role, primarily for post-operative follow-up and triage, mirroring Australian OMS experiences that identified willingness to continue telehealth but also persistent barriers related to patient needs, diagnostic accuracy and postoperative monitoring [21,22], as well as North American evidence on the accuracy of telemedicine consultations in OMS [23]. Barriers included digital literacy in elderly rural patients and medico-legal uncertainty in early 2020 [41], while moral concerns regarding the continuation of routine dental care during the pandemic were also emphasized in the dental literature [42].

The present findings support several practical considerations for future public-health disruptions. First, maintaining uninterrupted access to preventive dental care may reduce delayed presentations and advanced oro-facial infections. Second, protected oncological and urgent OMS pathways should be preserved during major healthcare restrictions. Third, routine tele-triage and streamlined perioperative protocols may help maintain service continuity and improve post-crisis efficiency. Finally, cleaned ICD-10 case-complexity metrics may provide a simple supplementary indicator of delayed presentation during healthcare-system disruption.Our results align with those of Pabst et al. [4] and Figueiredo et al. [1] in terms of volume reduction and with those of Omari et al. [13] and Dudde et al. [9] in terms of complexity increase. Multicenter European studies also reported changes in the epidemiology and etiology of maxillofacial trauma during the COVID-19 pandemic and lockdown periods [40,43].

They differ in two notable ways. First, the speed of our post-pandemic recovery (+10.1% in 2024) is superior to what Colin et al. documented in France [14], supporting the possibility that the Romanian public emergency system benefits from a centralized catchment structure. Second, the distribution of severe infections shifted toward L02/L03 codes rather than the classical K12 abscess increase reported in Germany, probably reflecting a local pattern of late presentation and progression through deep cervical fascial planes.

### Limitations

This study has several limitations. (i) It is a single-center retrospective analysis; although the clinic serves ≈2.5 million inhabitants, the results do not permit generalization at the national level. (ii) Outcomes such as 30-day readmission, surgical-site infection, return to theatre and long-term oncological follow-up are not captured by the current inpatient database and were not analyzed. (iii) ICD-10 coding is inherently dependent on the accuracy of the discharging physician and may underestimate rare complications. (iv) We cannot directly measure workforce burnout or PPE tolerability. (v) The comparison between “pandemic” and “post-pandemic” is necessarily affected by pandemic-era behavioral changes that persist. (vi) The progressive redirection of pediatric OMS admissions to the regional “Louis Țurcanu” Children’s Emergency Hospital from October 2021 onwards shapes the age composition of the post-pandemic dataset and should be borne in mind when interpreting any age-related comparison. (vii) The D37–D48 “uncertain behavior” surge cannot be fully decomposed into real biopsy backlog versus coding-behavior drift without external histology linkage (16.2% of D37–D48 patients were subsequently re-coded with a definitive C or D10–D36 diagnosis; the remaining 83.8% were not). (viii) We treat the catchment population as the sum of the five covered counties; small inter-year population shifts from INS Tempo Online have been incorporated into Supplementary Table S2, but cross-border or internal-migration effects below the county level cannot be resolved. Despite these limitations, the strength of the dataset—seven full calendar years, more than 11,000 consecutive admissions, a unified ICD-10 coding scheme and uninterrupted emergency activity—provides a level of internal consistency that is difficult to match with multi-center or survey-based studies.

## 5. Conclusions

In a tertiary Romanian emergency hospital serving approximately 2.5 million inhabitants, the SARS-CoV-2 pandemic caused a severe but transient reduction in oral and maxillofacial surgery inpatient activity, with a nadir of −76% in April 2020 and an average reduction of approximately 40% during 2020–2022. This reduction was accompanied by a significant redistribution of the case mix, including a marked reduction in elective benign-neoplasm admissions, a relative increase in D37–D48 diagnoses, and a modest increase in cleaned case-complexity indicators during the pandemic period. Length of hospital stay did not increase during the pandemic and became significantly shorter in the post-pandemic period after multivariable adjustment for demographics and case mix. By 2024, inpatient activity had recovered to and exceeded the pre-pandemic baseline (+10.1% raw counts; +16.2% population-adjusted).

These findings suggest that a centralized public emergency OMS service can recover rapidly after major healthcare disruption, while also highlighting the importance of maintaining pandemic-preparedness protocols, protected oncological pathways, and continuity of preventive dental care during future public-health crises.

## Figures and Tables

**Table 1:** Demographic characteristics of OMS admissions by period (2018–2024).

Variable	Pre-Pandemic (2018–2019)	Pandemic (2020–2022)	Post-Pandemic (2023–2024)	*p*-Value
Admissions, *n*	4074	3637	3917	–
Unique patients, *n*	2916	2594	2920	–
Age, mean ± SD (y)	52.3 ± 19.2	52.7 ± 19.2	51.7 ± 19.2	0.073 ^1^
Age, median (IQR) (y)	55 (37–68)	55 (38–67)	54 (35–68)	–
Male, *n* (%)	2262 (55.7)	2094 (57.6)	2204 (56.3)	0.23 ^2^
Female, *n* (%)	1796 (44.3)	1539 (42.4)	1711 (43.7)	–
Pediatric (5–14 y), *n* (%)	37 (0.9)	24 (0.7)	3 (0.1)	<0.001 ^2^
From Timiș county, *n* (%)	1894 (46.5)	1517 (41.7)	1603 (40.9)	<0.001 ^2^
Readmission (≥2/patient), *n* (%)	1158 (28.4)	1043 (28.7)	997 (25.5)	0.002 ^2^

^1^ Kruskal–Wallis test; ^2^ χ^2^ test of independence.

**Table 2:** Case-mix distribution by primary ICD-10 category and period.

Diagnostic Category	Pre-Pandemic *n* (%)	Pandemic *n* (%)	Post-Pandemic *n* (%)	*p*-Value
Severe odontogenic/oro-facial infections (K12.2, K10.2, L02.0, L03.2, K04.x)	1089 (26.7)	791 (21.7)	954 (24.4)	<0.001
Maxillofacial trauma (S02, S03, T88.x)	414 (10.2)	403 (11.1)	325 (8.3)	<0.001
Malignant neoplasms (C00–C80)	184 (4.5)	177 (4.9)	78 (2.0)	<0.001
Benign neoplasms (D10–D36)	292 (7.2)	72 (2.0)	106 (2.7)	<0.001
Neoplasms of uncertain behavior (D37–D48)	638 (15.7)	1001 (27.5)	992 (25.3)	<0.001
Other/unspecified	1457 (35.7)	1193 (32.8)	1462 (37.3)	0.002
Total	4074 (100)	3637 (100)	3917 (100)	–

Global Pearson χ^2^ test for overall diagnostic-category distribution across study periods: χ^2^ = 406.9, *p* < 0.001.

**Table 3:** Length of hospital stay (LOS) by period.

LOS Metric	Pre-Pandemic	Pandemic	Post-Pandemic	Statistical Test
N admissions	4074	3637	3917	–
Mean ± SD (d)	4.23 ± 3.50	3.92 ± 3.47	3.28 ± 2.58	H = 151.9; *p* < 0.0001
Median (IQR) (d)	3.07 (2.00–5.85)	3.06 (2.03–4.97)	2.22 (1.95–4.10)	(Kruskal–Wallis)
Total bed-days	17,240	14,242	12,834	–
Pre vs. Pandemic	–	–	–	U = 7,535,406; *p* = 0.19
Pandemic vs. Post	–	–	–	U = 8,100,844; *p* < 0.0001
Pre vs. Post	–	–	–	U = 9,103,426; *p* < 0.0001

**Table 4:** Case complexity, COVID-screening coding and comorbidity codes per period.

Variable	Pre-Pandemic	Pandemic	Post-Pandemic	*p*-Value
ICD-10 codes/admission, mean ± SD	3.44 ± 2.30	4.08 ± 2.42	3.37 ± 2.27	<0.0001 ^1^
ICD-10 codes/admission, median (IQR)	3 (2–5)	4 (3–6)	3 (2–5)	–
>3 codes/admission, *n* (%)	2010 (49.3)	2342 (64.4)	1779 (45.4)	<0.0001 ^2^
Z11.5 (SARS-CoV-2 screening), *n* (%)	0 (0.0)	2051 (56.4)	475 (12.1)	<0.0001 ^2^
R63.3 (feeding difficulty), *n* (%)	1688 (41.4)	1932 (53.1)	1436 (36.7)	< 0.0001 ^2^
I10 (hypertension), *n* (%)	1183 (29.0)	1129 (31.0)	1276 (32.6)	0.002 ^2^
In-hospital deaths, *n* (%)	3 (0.07)	7 (0.19)	3 (0.08)	0.15 ^3^

^1^ Kruskal–Wallis; ^2^ χ^2^ test; ^3^ Fisher’s exact test.

## Data Availability

The de-identified dataset is available from the corresponding authors upon reasonable request, subject to a data-transfer agreement compliant with the EU General Data Protection Regulation (Regulation 2016/679) and Romanian Law 190/2018.

## Supplementary Materials

The following supporting information can be downloaded at: https://www.mdpi.com/article/10.3390/medicina62061129/s1. Figure S1: Heatmap of monthly admissions per ICD-10 chapter, 2018–2024. Figure S2: Interrupted time-series model for monthly admissions. Table S1: Full list of ICD-10 primary codes per period. Table S2: County-level distribution of admissions per period.
