# Supplementary figures and images for: Impact of the SARS-CoV-2 Pandemic on Oral and Maxillofacial Surgery Activity: A Seven-Year Retrospective Study from a Romanian Emergency Hospital
Source: Medicina (Kaunas). 2026 Jun 10;62(6):1129. doi: 10.3390/medicina62061129 (PMC13303528; doi:10.3390/medicina62061129)

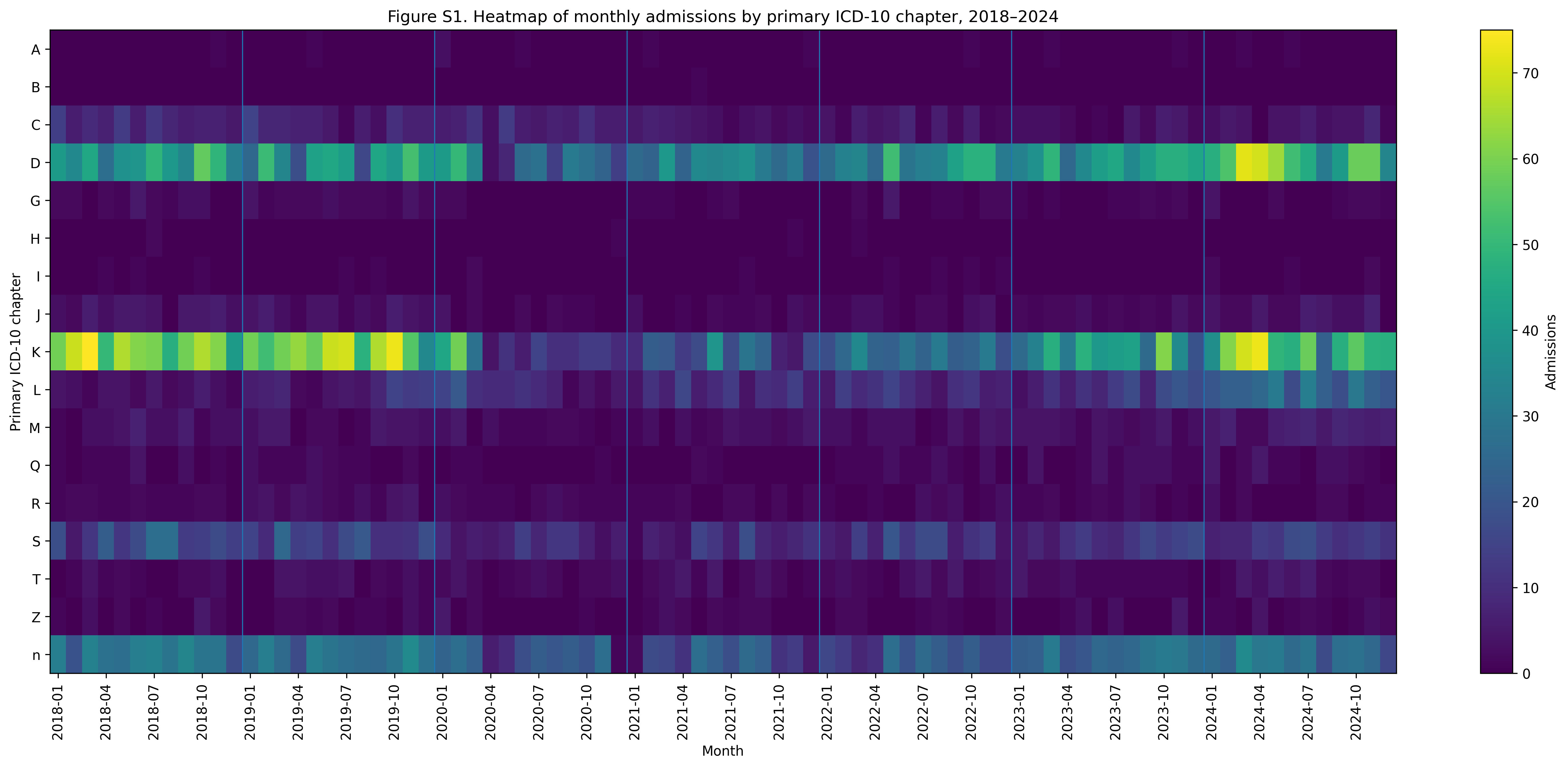

Supplement: Supplementary file 1 [file medicina-62-01129-s001.zip › Figure_S1.png]

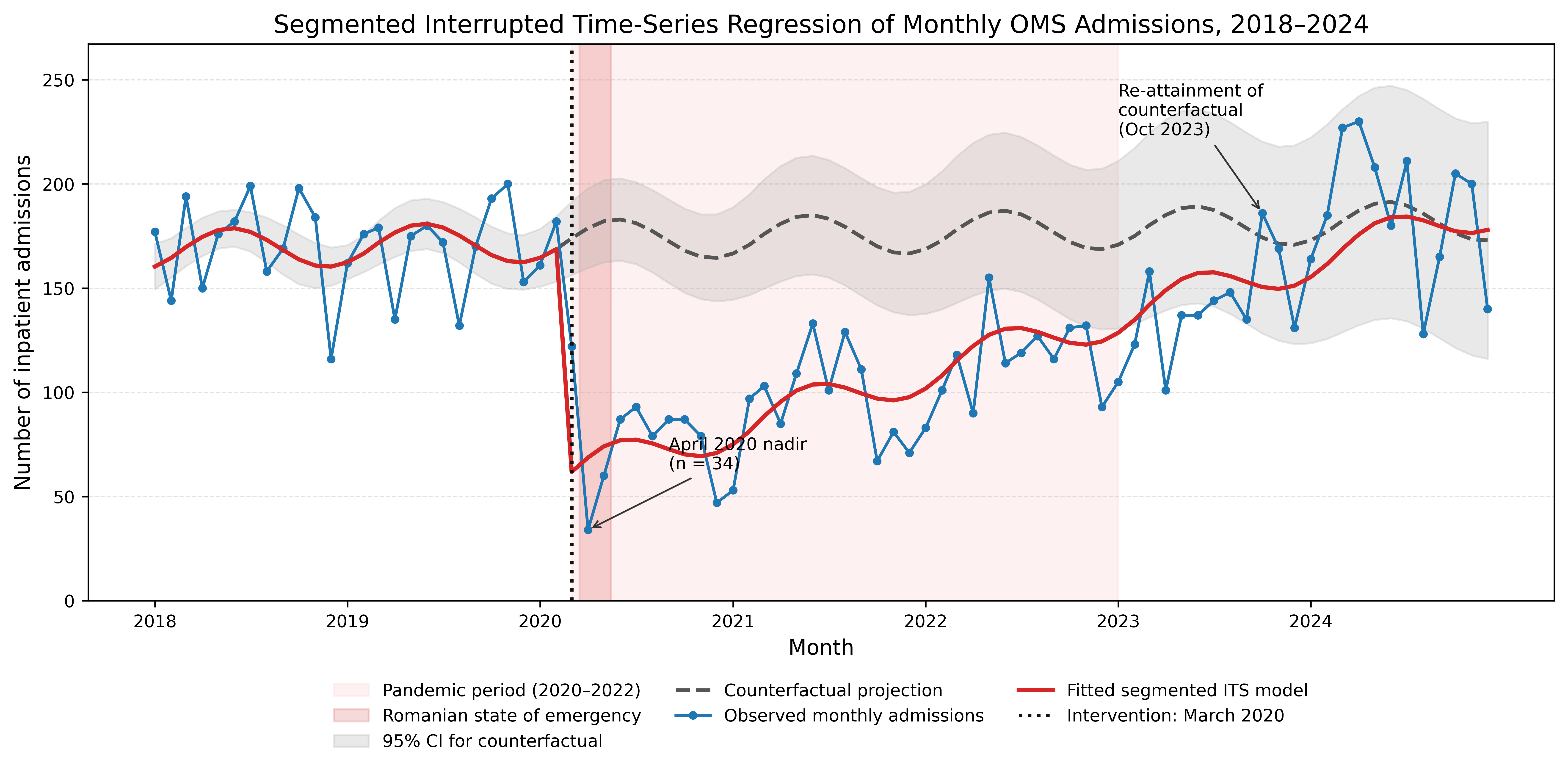

Supplement: Supplementary file 1 [file medicina-62-01129-s001.zip › Figure_S2.tiff]
